# Cutting-Edge Advances in Electrochemical Affinity Biosensing at Different Molecular Level of Emerging Food Allergens and Adulterants

**DOI:** 10.3390/bios10020010

**Published:** 2020-02-06

**Authors:** Susana Campuzano, Víctor Ruiz-Valdepeñas Montiel, Verónica Serafín, Paloma Yáñez-Sedeño, José Manuel Pingarrón

**Affiliations:** Departamento de Química Analítica, Facultad de CC. Químicas, Universidad Complutense de Madrid, E-28040 Madrid, Spain; vrvmontiel@ucm.es (V.R.-V.M.); veronicaserafin@ucm.es (V.S.); yseo@quim.ucm.es (P.Y.-S.)

**Keywords:** electrochemical affinity biosensors, allergens, gluten, adulterants, multiplexing

## Abstract

The presence of allergens and adulterants in food, which represents a real threat to sensitized people and a loss of consumer confidence, is one of the main current problems facing society. The detection of allergens and adulterants in food, mainly at the genetic level (characteristic fragments of genes that encode their expression) or at functional level (protein biomarkers) is a complex task due to the natural interference of the matrix and the low concentration at which they are present. Methods for the analysis of allergens are mainly divided into immunological and deoxyribonucleic acid (DNA)-based assays. In recent years, electrochemical affinity biosensors, including immunosensors and biosensors based on synthetic sequences of DNA or ribonucleic acid (RNA), linear, aptameric, peptide or switch-based probes, are gaining special importance in this field because they have proved to be competitive with the methods commonly used in terms of simplicity, test time and applicability in different environments. These unique features make them highly promising analytical tools for routine determination of allergens and food adulterations at the point of care. This review article discusses the most significant trends and developments in electrochemical affinity biosensing in this field over the past two years as well as the challenges and future prospects for this technology.

## 1. Introduction

Food allergies are caused by IgE- or cell-mediated humoral immune responses to the exposure to certain life-threatening antigens. These allergies currently represent one of the major food safety concerns in industrialized countries and affect 1–10% of the global population, with higher prevalence in children. Food allergies are generally controlled by avoiding contact/consumption of allergenic foods by sensitized persons or by treating symptoms. However, the first option may be particularly complex, considering the possible presence of hidden allergens, exposure to allergens from adulterated products, and cross-contamination [1]. Moreover, food adulteration affects product quality and processing, and can pose health risks and economic and confidence problems for consumers [2,3]. 

Thus, with the main objective of helping the food industry, food manufacturers and suppliers, and protecting consumer rights avoiding unfair practices and competition in the market, reliable analytical methodologies for the detection of allergens and adulterations in food have been developed and marketed. However, there is still a need to develop methods suitable for routine point-of-care (POC) analyses in a simpler and more cost-effective manner [4]. In this context, electrochemical affinity biosensors, due to their low cost, simplicity of use and compatibility with portability and automation, are a particularly interesting alternative. 

Here we review the recent developments in electrochemical biosensing strategies for the determination of food allergens and adulterants. Some nice reviews and book chapters have been published on this topic [4,5,6,7,8,9,10,11]. Therefore, we limit our discussion to the state-of-the art by highlighting representative and innovative methods reported since 2017. 

## 2. Food Safety: Allergens and Adulterations

Food allergy, one of the major health problems affecting millions of people worldwide, especially in industrialized countries, is an abnormal immune response that arises after eating certain types of food and affects approximately 4% of the adult population with a higher prevalence among children (6–8%).

This immune hypersensitivity is mediated by allergen-specific immunoglobulin E (IgE), usually against certain dietary proteins or glycoproteins (antigens), by cells or by a mixed IgE/cell mechanism. While IgE-mediated hypersensitivity is associated with rapid symptom development (type I or immediate hypersensitivity), usually within minutes to 2 h after exposure to a specific allergen, cell-mediated reactions develop over hours or days (type IV or delayed hypersensitivity) [4].

Although very small amounts of allergens (from less than 1 mg to a few grams) are required to cause an allergic reaction in a sensitized person, clinical symptoms depend on the allergen dose and the sensitivity of the individual, and may also change over time and vary geographically according to exposure to allergens [4].

The increased incidence of hypersensitivity to food allergens may be due to a combination of factors such as globalization, cross-contamination, lack of good manufacturing practices, etc. It is also important to note that the variability in the number and intensity of symptoms derived from these hypersensitivities, together with a lack of knowledge of why certain dietary proteins are more likely to trigger an allergic response, complicate the diagnosis and treatment of food allergies. 

Although there are several treatments, there are still no currently accepted therapeutic approaches for food allergy, which is usually controlled by avoiding contact with allergens or treating symptoms once they appear. With this in mind, food labeling plays a crucial role in providing information to sensitized consumers and enabling to implement a successful prevention strategy [4].

Although more than 160 food materials are considered allergenic compounds, European legislation requires that 14 allergenic food ingredients need to be clearly labeled on the food product. In addition, it is known that approximately 90% of food allergies worldwide are caused by eight major allergens: eggs, milk, crustaceans, shellfish, fish, peanuts, nuts, soybeans, and wheat, which must be clearly identified on product labels by food manufacturers (FALCPA 2004, Public Law 108–282, Title II). On the other hand, the European Union (Directive 2007/68/EC) requires a mandatory declaration of the following allergenic foods: crustaceans, eggs, fish, peanuts, soybeans, milk, nuts, celery, mustard, sesame seeds, lupins, molluscs and their respective products. Cereals containing gluten and sulphur dioxide/sulphites are also included in the mandatory reporting list, as they are responsible for other adverse food reactions (coeliac disease or gluten intolerance/sensitivity and idiosyncratic reactions, respectively) in some individuals [4].

However, it is important to be aware that inadvertent contamination of foods with allergens that do not appear on the food label can occur during any stage of the food chain. Therefore, allergy sufferers may be accidentally exposed to allergenic proteins through the consumption of products that are supposed to be free of allergens. Undeclared allergenic substances may be present in a food product through adulteration, fraud or uncontrolled cross-contamination, posing a real threat to the health of allergic consumers. Cross-contamination can occur during food manufacturing due to the use of shared equipment with insufficient cleaning or during transfer or storage processes. This is the reason why many industrial companies have separate production lines for different types of products. However, it can be difficult for the food industry, food manufacturers and restaurant chains to ensure that a food product is free of allergens, especially when different types of products are produced and stored in the same facilities. Accordingly, food industry often opts for a preventive labeling to alert the possible presence of hidden allergens due to these cross-contamination processes. This danger of unexpected exposure to hidden allergenic ingredients, mainly in processed foods, has led to a high demand for the development of high sensitive allergen detection tools enabling regulators and food manufacturers to assess and manage the risk of food allergen contamination [12]. 

Food fraud, on the other hand, is a collective term used to encompass the deliberate and intentional substitution, addition, tampering, or misrepresentation of food, its ingredients, or packaging to obtain an economic benefit, and involves a loss of consumer confidence. Food fraud can also be viewed as a public health problem if involved undeclared toxic substances or allergens. One of the types of food fraud that accounts for around 3% of total food infringements is adulteration, where the addition of foreign, unsuitable or poor quality ingredients to a product is considered to be motivated by economic reasons. Currently, a large number of foods (milk, olive oil, honey, saffron, coffee, orange and apple juices and meat products [3]) may be subject to such irregularities. Although some adulterations may pose a health risk, such as the adulteration of milk with milk of other animal origin, due to the sensitivity of certain individuals to proteins of a certain animal origin, others, such as the presence of different types of meat in other animal products, do not usually represent a health risk. However, in all cases it is up to consumers to choose according to their lifestyle, customs or beliefs, which is what they consume.

## 3. Conventional Methods to Determine Food Allergens and Adulterants

The determination of allergens and adulterants in food can be a difficult task, due to their presence generally in vestigial amounts, together with natural matrix interference. In addition, very often, and generally as a consequence of the foods thermal processing, changes in the biophysical and immunological properties of allergic proteins may occur which may affect the allergenicity (increasing or decreasing it) and hinder its determination [4,13].

The methods reported for the determination of allergens and adulterants can be divided into two major groups: immunological methods and DNA-based assays. Another common classification is in direct or indirect methods, aimed at the determination of the allergen/adulterant itself or of biomarkers characteristic of its presence. 

In general, immunological methodologies rely on the specific binding between the epitopes of the target molecule and a specific immunoglobulin [4]. Although, ideally, detection limits (LODs) for allergenic proteins should be between 1 and 100 mg kg^−1^ of food, depending on the sample analyzed and its allergenicity to protect more sensitized consumers [4], lower values are demanded to highly allergenic foods such as peanuts (with which subjective symptoms have been reported even with doses as low as 100 μg [14,15]). Moreover, it should be considered that, apart from the effect of food processing on the target protein structure, several allergenic foods contain multiple allergenic proteins that may vary in abundance, which may interfere with detection [4].

Enzyme-linked immunoabsorption assay (ELISA) is the most common immunological method used for the semi-quantitative or quantitative determination of allergens in food industries and official food control agencies due to its high sensitivity (1–25 ppm), accuracy, simple handling, and standardization potential [4]. 

A large number of commercial ELISAs kits are currently available and for a wide variety of food allergens [13], involving mainly sandwich but also competitive type formats. As disadvantages, these tests can be slow (up to 3.5 h), are prone to the appearance of cross-reactions, they do not provide fully comparable results due to the use of different immunoreagents [16] and require relatively expensive instrumentation (ELISA plate readers), hardly portable and miniaturizable. It should also be noted that only a limited number of kits have been validated by the international Association of Analytical Communities (AOAC).

Although less frequently used, other immunological methods such as lateral flow assay (LFD), dipsticks, rocket immunoelectrophoresis and dot-immunoblotting have been reported. However, most of these methods are semi-quantitative or only qualitative [4,12]. Other methods involved previous separation of the allergic proteins by gel electrophoresis, capillary electrophoresis or high performance liquid chromatography (HPLC). 

Other recent approaches reported for the detection of allergens at the protein level include new peptidomics and proteomics methods, flow cytometry and immunochromatography [1], surface plasmon resonance (SPR) [17] and cell-based biosensors [18].

Unlike methods based on the direct detection of adulterants and protein allergens, DNA-methods are based on their indirect determination by targeting a characteristic fragment of the gene that encodes their expression generally amplified using polymerase chain reaction (PCR) technologies [4,12]. The PCR-based methods can provide qualitative, semi-quantitative (PCR-ELISA) or quantitative real-time PCR (RT-PCR) analyses and are advantageous for the simultaneous detection of multiple analytes. However, they are not appropriate for food allergens that contain a large level of protein and a low abundance of DNA, such as eggs, and can sometimes be controversial, as processing may affect nucleic acids and proteins differently. In addition, allergen/adulterant levels encoding DNA are not always correlated with the presence of the allergen/adulterant, especially when foods are fortified with purified proteins. These PCR methods are considered ideal tools to be used complementary to the immunological methods [4].

Mass spectrometry and other chromatographic techniques have also been used as confirmatory tools for the unequivocal identification and/or characterization of food allergens in different products [4].

Importantly, despite steady progress, these methods still do not meet the current needs of POC technology for the detection of food allergens and adulterants in terms of simplicity, timeliness, cost and portability [1,19].

Although their use outside the research environment is still limited, biosensors have recently shown to be an excellent alternative for such purpose and new strategies are constantly being reported involving novel affinity bioreceptors, transducers and amplification strategies that improve the resulting sensing performance [12,20]. Antibodies against food allergen/adulterant specific proteins and synthetic peptide or oligonucleotide (of DNA or RNA nature, linear, switching-based or aptameric) probes targeting characteristic fragments of the allergen/adulterant encoding gene or protein biomarkers by efficient hybridization or affinity reactions, respectively, can be used as recognition receptors. It is important to note that the vast majority of biosensors described to date for the determination of food allergens and adulterants are immunosensors. 

In the particular case of food allergen analysis, sample preparation is a crucial step. One of the greatest challenges to avoid false positives and negatives is to extract successfully the respective allergenic protein or DNA from a complex matrix that contains a number of interferences that may significantly affect the result of the method. In addition, certain extraction conditions, such as temperature or pH, can significantly affect product stability. It is also important to bear in mind that the sample preparation depends on the type of affinity sensor used for the determination, and, in general, it is slightly more complex for nucleic acid biosensors than for immunosensors [4].

Both optical and electrochemical biosensors have proven to be suitable for allergen detection. However, electrochemical biosensors are a particularly attractive alternative to meet the requirements currently demanded by the field of food safety for on-site, rapid and affordable analyses in outside laboratory environment by unskilled personnel [9]. 

## 4. Electrochemical Affinity Biosensors for the Determination of Food Allergens and Adulterants

**Figure 1:**
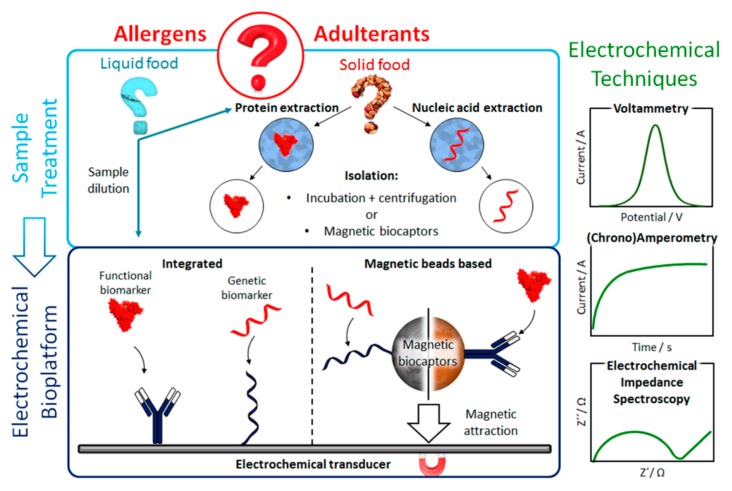
Schematic display of the fundamentals (target biomarker extraction and electrochemical detection) involved in the construction of electrochemical biosensors for the determination of food allergens and adulterants at genetic or functional level using integrated or magnetic beads (MBs)-based strategies.

Electrochemical biosensors (Figure 1) exhibit several advantages over optical biosensors in terms of cost, miniaturization potential, portability and analytical accuracy for complex food matrices [1,12,19], which make them particularly interesting for implementation in POC devices. Moreover, signal detection, based on electrochemical reactions, is fast, scalable, susceptible to multiplexing and suitable for compact electronic devices [21]. The most common electrochemical techniques for the determination of allergens and food adulterations are voltammetry, amperometry and impedimetry. 

(Bio)nanotechnology has allowed a remarkable improvement in the analytical performance of electrochemical biosensors. Novel bioreceptors and bioassay configurations, implemented in integrated formats or involving magnetic particles (MBs) (Figure 1) have been designed and explored. Moreover, important progress has been made in the manufacture of versatile transducers with improved properties. In addition, the synthesis and modification of nanomaterials (mainly used in this field as electrode modifiers), the application of attractive surface chemistries (such as diazonium salts or thiols), and the use of amplification strategies involving commercial multienzymatic reagents, enable electrochemical biosensors to meet the requirements of simplicity, speed, sensitivity and selectivity demanded by routine control of food allergens and adulterants. Furthermore, the versatility exhibited by electrochemical biosensing for the multidetermination of analytes at different molecular levels or with very different concentration ranges as well as its applicability to complex food matrices with minimal pre-treatments, are particularly attractive characteristics also in this field. 

A very useful strategy in electrochemical biosensing for food safety is the combined use of MBs with screen-printed electrodes (SPEs). SPEs are mass and inexpensively produced from a variety of materials, in different geometries and in miniaturized and multiplexed formats and allow working with very small sample volumes [5,22]. 

MBs are a powerful and versatile tool employed in biosensing because they facilitate efficient target analyte retrieval and concentration, minimize matrix effect, reduce largely the assay time and make analytical procedures more compatible with higher sample throughput and automation [23,24,25,26].

As in other biosensing fields, the use of nanostructured electrode surfaces, most commonly with carbon nanomaterials [27,28] and metal nanoparticles [29,30], has contributed significantly to the better performance of electrochemical biosensing for food analysis. The use of nanomaterials as electrode modifiers has been exploited mainly for: (i) enhancing the electrochemical response due to the excellent behavior as electrical conductors of many nanomaterials; (ii) creating suitable environments for the immobilization of bioreagents with extended lifetime and (iii) developing no-wash electrochemical biosensors [31].

Immunosensors are the most widely used biosensors to date in this field. However, the well-known antibodies limitations such as their in vivo production, low stability and possible cross-reactivity with non-target compounds of similar structure, has led to explore the use of synthetic receptors such as aptamers [32,33,34], peptides [34], nucleic acids and oligonucleotide-switching probes [35]. 

The biosensors recently developed for the determination of DNA fragments characteristic of allergens or adulterants try to replace the conventional PCR amplification by other shorter strategies (Express PCR [36]) or with simpler implementation in POC devices (not requiring a thermocycler) using multienzyme bioreceptors [37,38,39].

In the following two subsections cutting-edge advances in electrochemical affinity biosensing for the determination of well-known or emerging food allergens or adulterants as well as for gluten, are critically discussed through representative works reported since 2017. Table 1 and Table 2 (placed at the end of the corresponding section) summarize the interesting features of the selected immunosensing and nucleic acid-based biosensing methods, respectively. Other food intolerances caused by substances that show pharmacological activity, such as vasoactive amines, and food adulterations with antibiotics are considered out of the scope of this review focused on electrochemical affinity biosensors proposed for the determination of food allergens and adulterants at the genetic and protein level.

### 4.1. Electrochemical Immunosensing Methods

Most of the electrochemical immunosensing strategies reported recently are focused to the determination of allergen proteins and much less to adulterants (see Table 1). These strategies are implemented both in integrated formats and on MBs surface and in most cases used SPEs as electrochemical transducers. Sandwich assays involving a pair of specific antibodies capable of recognizing different epitopes of the target allergen [21,40,41,42] or the same antibody (Ab), unmodified as capture and labeled with horseradish peroxidase (HRP) as detector antibodies, respectively, [43,44] are mostly employed. Nevertheless, strategies involving direct-type immunoassays by immobilizing the antibody or the antigen have also been described. The reported methods were developed for the determination of the main allergenic proteins of peanuts, eggs, gluten, milk and shrimps and for the detection of adulterations in milk with milk of other animal origin or with colostrum, allowing the determination of the target analyte at the level of a few ng mL^−1^ or mg kg^−1^. It is remarkable the low LOD reached (47 pg mL^−1^) in the strategy developed by Angulo-Ibáñez et al. [41]. The high sensitivity can be attributed to the binding of multiple molecules of secondary antibodies to each single primary antibody [45,46]. This enhances the sensitivity vs. the use of HRP-labeled DAb or of four-multivalent streptavidin (Strep)-based enzymatic conjugates which can cause cross-reaction between different biotinylated detector antibody (bDAb) molecules [47,48]. 

It is important to note that the strategies developed for the determination of bovine, ovine and caprine immunogobulins (IgGs) [43] and ovomucoid [44] are the first electrochemical bioplatforms reported for the determination of such analytes. Many of the reported strategies have demonstrated their usefulness for the quantification of the target analytes in raw or processed complex food matrices. The reported methods require between one and four steps to perform the determination (without counting detection and sample preparation steps and starting from the modification of the electrode or MBs with the bioreceptor) and assay times between 30 min and 4 h. Furthermore, the immunosensing platforms developed in the last two years involve commonly label-based strategies and make use of enzymes, MBs or nanomaterials (gold nanoparticles, AuNPs, single-walled carbon nanotubes, SWCNTs, and carbon nanotubes, CNTs) as electrode modifiers to achieve the high sensitivity demanded. 

The most sensitive integrated immunoplatforms have been implemented on nanostructured substrates (AuNPs-SPCE, SWCNTs-Au-Cr deposited silicon wafer and carbon nanofibers (CNFs)/SPCE). The preparation of an immunosensing platform onto paper microzone is also remarkable. (Chrono)amperometry is commonly used to monitor the enzymatic reduction (HRP) of H_2_O_2_ mediated by 3,3′,5,5′-tetramethylbenzidine (TMB) or hydroquinone (HQ). Nevertheless, linear square voltammetry (LSV) measurement of the metallic silver oxidation deposited on the electrode surface in the presence of AgNO_3_ after 3-indoxyl phosphate (3-IP) hydrolysis by alkaline phosphatase (AP) has also been exploited [49]. 

Because of the attractive analytical performance in terms of sensitivity, simplicity and testing time, the immunosensing strategy developed by Benedé et al. [44] for the determination of egg traces through ovomucoid, a white protein highly resistant to denaturation, aggregation and proteolytic degradation can be remarked [50]. The method involves a single 30-min incubation step and provides a LOD of 0.1 ng mL^−1^, 610 times lower than that of the commercial ELISA kit using the same immunoreagents, and in a 3 times shorter analysis time. It was applied to the analysis of wheat flour and baked bread supplemented with lyophilized egg at trace levels with ability to detect 0.17 and 0.13 ppm of egg in raw (wheat flour) and cooked (baked bread) samples, respectively. 

**Figure 2:**
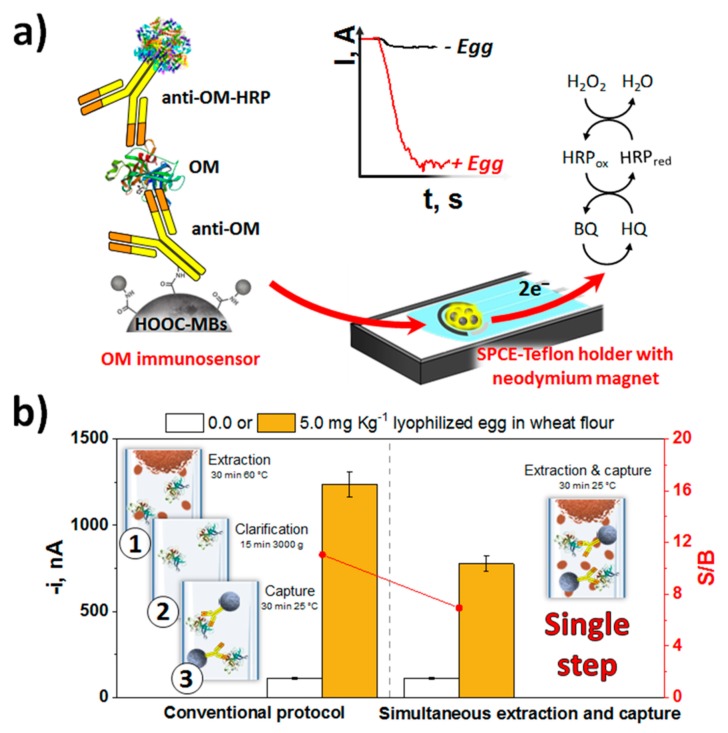
(**a**) Schematic display of the sandwich immunoassay involving MBs developed for the determination of ovomucoid protein and the use of the same antibody, unmodified for capturing the target allergenic protein, and conjugated with HRP for its enzymatic labeling. (**b**) Comparison of the amperometric responses obtained with the developed immunosensor for wheat flour samples unspiked or spiked with 5.0 ppm of lyophilized egg by performing the proteins extraction and the selective capture of ovomucoid by CAb-MBs in separate or single steps. Figure drawn based on [44].

These values were four orders of magnitude lower than those provided by commercial ELISA kits and those considered relevant for the determination of allergens (10 mg Kg^−1^ [51,52]). Importantly, it was possible to detect only 5 ppm of egg by a simple incubation of the CAb-MBs in turbid samples with a high content of suspended solid matter to perform the direct and efficient ovomucoid capture (Figure 2b). This much simpler and faster protocol would be suitable for application at the point of attention since it did not require the application of centrifugation or high temperature processes for extracting the allergenic protein from the food matrix (both raw and processed samples).

A low LOD and the suitable practical applicability make the strategy proposed recently by Angulo-Ibáñez et al. [41] particularly interesting for the fish and seafood processing industry, where fish and seafood (both crustaceans and mollusks) are often manipulated in the same plant and eventually used in the preparation of diverse products. The method involved an immunoplatform using MBs with an excellent selectivity against mollusk tropomyosin (TPM) which allowed discrimination between crustaceans (shrimp) and mollusks (squid) as well as its determination both in raw and cooked samples.

Due to the fact allergens can be cross-reactive, the development of multiplexed strategies able to simultaneously detect different biomarkers is in high demand. These strategies can provide more and more reliable information with a single sample, saving time and money and giving confidence to the consumers that their food does not contain any undesired component derived from different sources or from the same source [4,53]. In this context, the immunoplatforms developed by Lee [21] and Pingarrón [43] groups for the simultaneous determination of 5 relevant allergens and 3 immunoglobulins of animal origin, respectively, are noteworthy.

**Figure 3:**
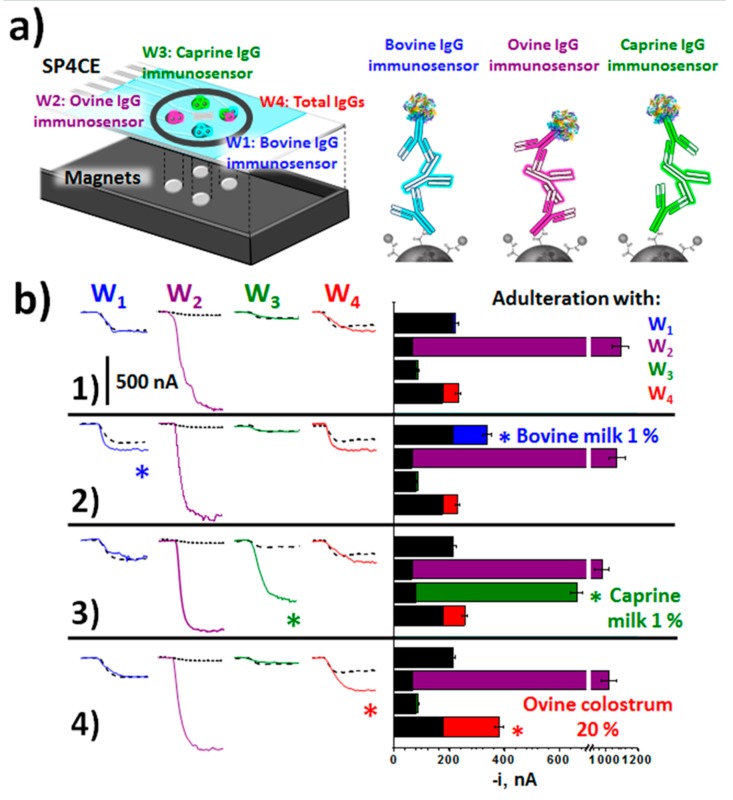
(**a**) Multiplexed MBs-based electrochemical immunoplatform for the detection of adulteration in milk by targeting bovine, ovine, caprine, and total IgGs in milk samples at SPC4Es. (**b**) Amperometric traces and measured currents (bars) in the analysis of 100 (W1–W3) or 10,000 times (W4) diluted raw sheep milk: unadulterated (**1**) or adulterated with 1.0% raw bovine milk (**2**), 1.0% raw caprine milk (**3**), and 20% ovine colostrum (**4**). For comparative purposes, amperometric traces recorded in the absence of bovine, ovine, and caprine IgGs (buffered solutions) are displayed as black striped bars. Figure drawn based on [43].

The method reported by Pingarrón and Campuzano’s group [43] allowed the identification of milk adulteration with milk from other animals or with colostrum. The method involved the preparation of immunosensing platforms for the detection of milk from cow, sheep and goat through the corresponding immunoglobulins G of these animal species, and their integration into a multiplexed electrochemical platform. Such immunoplatforms used sandwich immunoassay formats on MBs functionalized with carboxylic groups and involved the same unmodified antibody or conjugated with HRP as the capture and detection antibody, respectively, for each target IgG. Data in Table 1 shows as similar linear ranges and sensitivities were obtained for the determination of each target IgG, thus facilitating their integration in a multiplexed strategy. In addition, the achieved LODs were significantly lower than those claimed for most of the available ELISA kits, with an analysis time 2 to 4 times shorter. The developed immunoplatforms were able to unequivocally detect the presence of only 0.1% of cow’s, sheep’s or goat’s milk in milk of other species, a level ten times lower than that established by the European legislation. At present, there is not legislation on the levels of colostrum that may be contained in milk for marketing. Nevertheless, there is growing awareness of the drawbacks of its presence in the dairy industry, particularly for the manufacture of cheeses. The mentioned immunoplatform was also used to develop a method, based on the combination of individual batches of three magnetic immunoconjugates for the total detection of IgG bovine, ovine and caprine, which allowed the identification of colostrum in dairy products. In addition, the individual methodologies were employed to develop a multiplexed immunosensing platform with four working screen-printed electrodes (SP4CEs), for the screening, in a single determination, of the animal origin of a milk sample, the heat treatment undergone and the potential adulterations with milk from other animal species or colostrum. The protocol implied the magnetic capture of the immunocomplexes used for the detection of individual or total IgGs on each of the four electrodes of the SP4CEs (Figure 3a). Figure 3b displays the amperometric traces recorded and the results showing the usefulness of the multiplexed platform for screening unadulterated or adulterated milk samples in just 30 min, thus allowing its use for routine screening of milk samples in decentralized environments and by unskilled personnel. 

**Figure 4:**
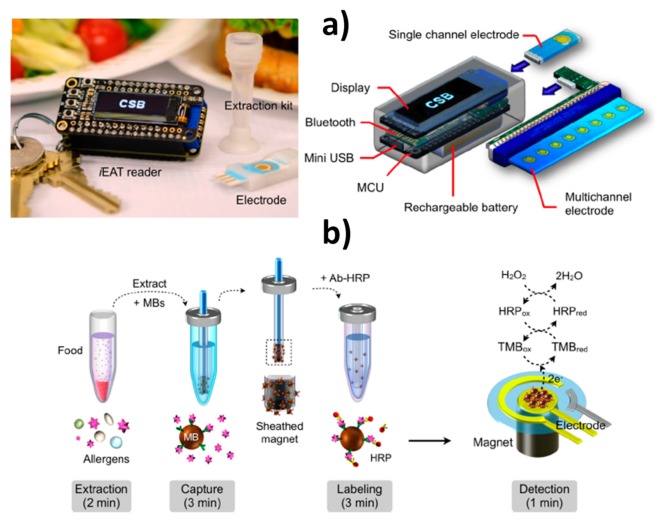
Integrated MBs-based immunosensing platform for on-site single or multiple food allergen detection. (**a**) iEAT system consisting of a pocket-size detector, an electrode chip, and a disposable kit for allergen extraction (left) and schematic of the pocket-size reader designed for standalone operation with its own display, rechargeable battery, and wireless communication module which allows to connect a single electrode or multiple electrodes through a card-edge connector. (**b**) Disposable kit to perform allergen extraction involving a sheathed magnet that collects the immuno-MBs. Once the MBs are modified with the HRP-labeled sandwich immunocomplexes they are dropped on the electrode and chronoamperometric transduction is performed in the presence of H_2_O_2_/TMB. Reprinted from [21], with permission.

Lin et al. reported a portable, termed integrated exogenous antigen testing (iEAT) with a degree of development close to its possible commercialization as a point-of-use technology for the rapid detection of five representative model food antigens in peanuts, hazelnuts, wheat, milk, and eggs [21]. The system consisted of a disposable allergen extraction device coupled with an inexpensive (<$40) electronic keychain reader with features both a single and a multichannel electrode able to measure eight allergens simultaneously (Figure 4). The system allowed antigen extraction and detection to be carried out in a short and actionable time at minimal cost (<10 min and <$4 for the entire assay) far surpassing the gold standard ELISA. The usefulness of this technology was demonstrated by surveying for the target allergens common foods (packaged staple food and desserts) and foods and gluten-free menus from local restaurants. These characteristics closely aligns the iEAT system with the World Health Organization (WHO) guideline for point-of-use ASSURED devices (affordable, sensitive, specific, user-friendly, rapid and robust, and no large electricity-dependent instruments) and to help consumers and other stakeholders (clinicians, food industries, and regulators) to enhance food safety.

### 4.2. Nucleic Acid-Based Biosensing Methods

The electrochemical nucleic acids biosensing methods reported in the last two years for the determination of food allergens or adulterants (whose main characteristics are summarized in Table 2) used different formats (direct, competitive and sandwich hybridization) and bioreceptors (synthetic sequences of different nature -DNA or RNA-, linear, aptameric or redox-tagged switching probes and selective antibodies for DNA/RNA heterohybrids). Using specific hybridization and/or affinity reactions, they have been applied to the direct or indirect determination of the allergens or adulterants by targeting proteins or DNA fragments that encode the expression of characteristic genes or are characteristics of the animal species, respectively. The indirect methods have been applied to the determination of: (i) amplicons obtained by Express PCR [36]; (ii) extracted genomic (gDNA) [57] or mitochondrial (mtDNA) DNA [37] without previous amplification or fragmentation and after a simple denaturation step; iii) mitochondrial raw lysates without previous mtDNA extraction [37]. Most of these methods used disposable electrodes and, to achieve the sensitivity and selectivity demanded by this type of applications were, in some cases, coupled to the use of nanomaterials or nucleic acid amplification strategies, as well as to the use of MBs and to multi-enzymatic detector bioreceptors [37,38,39]. 

Electrochemical biosensing platforms were prepared using sandwich-type hybridization methods on the surface of Strep-MBs for the identification of the presence and quantification of hazelnut [36,57] or tomato seeds traces through the detection of DNA fragments characteristic of the genes encoding the allergenic proteins Cor a 9 and Sola l 7, respectively. 

**Figure 5:**
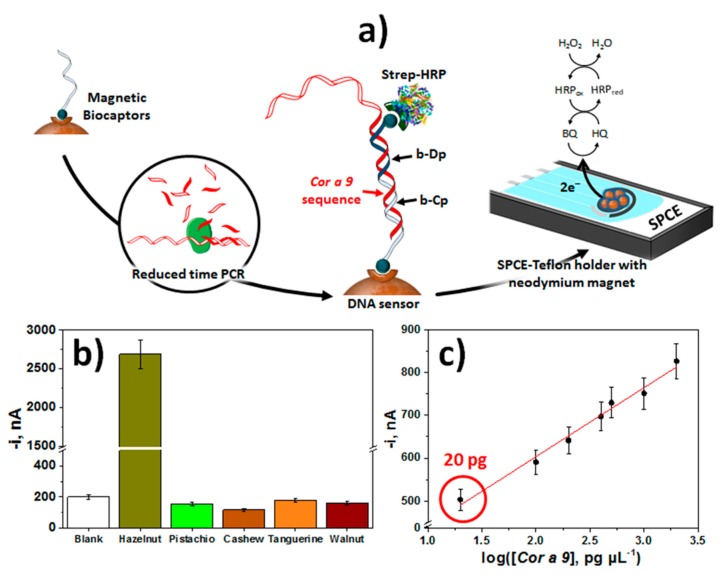
(**a**) Schematic display of the fundamentals involved in the construction of an electrochemical bioplatform using MBs for the detection of Express PCR amplified fragments specific to the hazelnut *Cor a 9* allergen coding sequence. (**b**) Amperometric responses provided by the developed bioplatform for 50-times diluted Express PCR amplicons obtained with gDNA extracted from hazelnut, pistachio, cashew, tangerine and walnut. (**c**) Dependence of the amperometric responses provided by the developed bioplatform for 50-times diluted Express PCR amplicons obtained using different amounts of hazelnut gDNA. Reprinted and adapted from [36], with permission.

The method developed for the detection of hazelnut was based on the use of synthetic biotinylated DNA capture and detector probes that hybridize contiguously with a characteristic fragment of the *Cor a 9* gene. Labeling of the resulting DNA homohybrid with Strep-HRP (Figure 5a) provided a LOD of 0.72 pM for the synthetic sequence in just a 15-min single incubation step starting from the preparation of the bCp-Strep-MBs [36]. Enhanced sensitivity for practical applications was achieved by means of an amplification strategy called reduced time PCR or Express PCR. This strategy reduced the amplification time by more than 1 h compared to conventional PCR and also improved the amplification efficiency. Through the analysis of the amplicons obtained from 100 bp, the method allowed the unequivocal detection of the presence of hazelnut (20 pg of gDNA) regardless of its variety (Figure 5b,c), which is a concentration 100 times lower than that can be detected using gel electrophoresis, and similar to that achieved using RT-PCR. 

Considering the growing demand of target amplification-free strategies, much easier to implement at the point of attention, the methodology reported for the detection of tomato seeds used a sandwich-type hybridization format involving two synthetic RNA probes of 30 nucleotides (nts) each, with the capture probe biotinylated, that hybridized contiguously with a characteristic 60 nts fragment of the *Sola l 7* encoding gene, and a commercial antibody (Ab_RNA/DNA_) capable of recognizing regions of only 6 bp in the formed RNA heterohybrid [57]. Due to the epitope size and the length of the heterohybrid, up to 10 DAb molecules could be bound by a single heterohybrid. The subsequent labeling of each DAb by several secondary antibodies conjugated with HRP [45,46] justified the high sensitivity achieved with this strategy without amplification of the target DNA. The method was able to detect the presence of tomato in 100 ng of gDNA extracted from this vegetable with only two incubation steps and in 90 min. 

**Figure 6:**
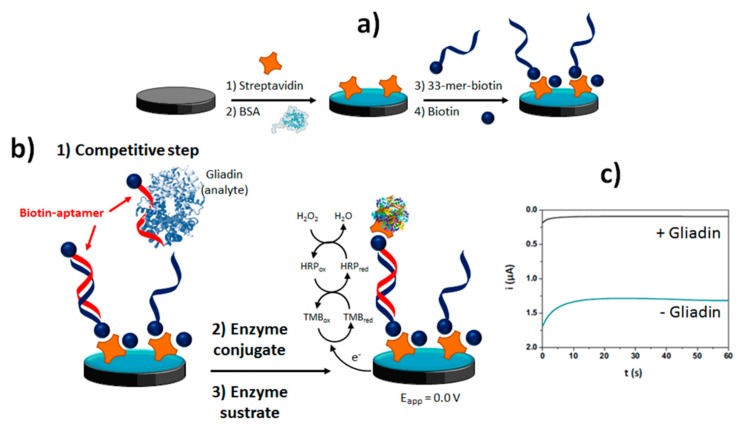
Competitive aptasensing method developed for the electrochemical determination of gluten: sensor fabrication (**a**) competitive assay (**b**) chronoamperometric transduction in the presence of H_2_O_2_/TMB (**c**) Figure drawn based on [16].

The methods developed making use of aptamers for the determination of protein allergens such as gluten or lysozyme is also noteworthy. Table 2 shows as a label-free aptasensor has been developed for the detection of lysozyme using a direct format implemented on electrodes nanostructured with AuNPs [33]. The methods developed for the detection of gluten, requiring a high sensitivity, involved competitive formats between gluten proteins (gliadin) and a synthetic biotinylated peptide immobilized on the surface of a SPCE (Figure 6a,b) [16] or Strep-MBs [34].

The biotinylated aptamers attached to the immobilized peptide was labeled enzymatically with Strep-HRP to perform chronoamperometric transduction with the H_2_O_2_/TMB system (Figure 6c). All these methods were applied to the determination of the target allergen in real samples (wine and food samples, respectively) within 1 h. 

**Figure 7:**
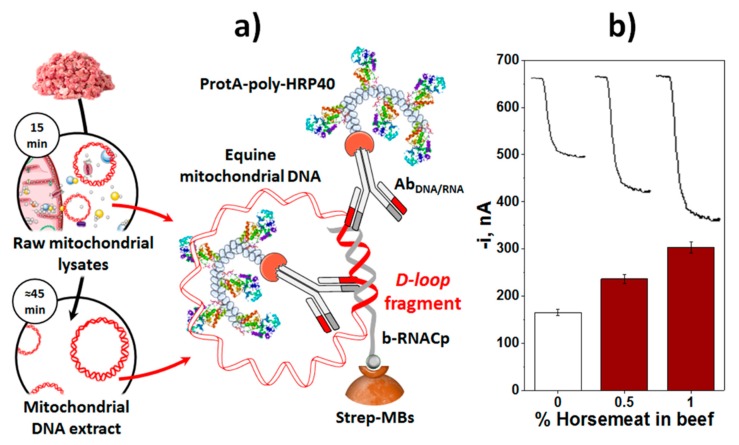
(**a**) Schematic display of the fundamentals involved in the amperometric MBs-based biosensor developed for the detection of horse mtDNA. (**b**) Amperometric traces recorded for 1:1 diluted raw mitochondrial lysates obtained from 3.0 g of mixtures prepared with beef and different percentages of horse meats. Figure drawn based on [37].

In the field of adulterations, relevant methods have been developed to detect the presence of horse meat in beef meat and melanin in milk. A fragment characteristic of the D-Loop region of horse mitochondrial DNA was selected for the specific detection of horse meat [37]. The developed assay involved a direct DNA/RNA hybridization implemented on the surface of Strep-MBs, and labeling of the resulting heterohybrid with a selective antibody (Ab_RNA/DNA_) and the bacterial antibody binding Protein A (from *Staphylococcus aureus*) able to recognize the Fc region of mammals IgGs [58,59] conjugated with a homopolymer containing 40 HRP molecules (ProtA-poly-HRP40, Figure 7a). This strategy detected the presence of horse meat, using amounts of mtDNA equal to or larger than 50 ng, with no need for fragmentation or amplification and discriminating the presence of only 0.5% of horse meat in beef meat (Figure 7b), the level required by European legislation, directly in raw mitochondrial lysates obtained from meat samples by a simple 15 min-centrifugation step, in a total assay time of 75 min. 

**Figure 8:**
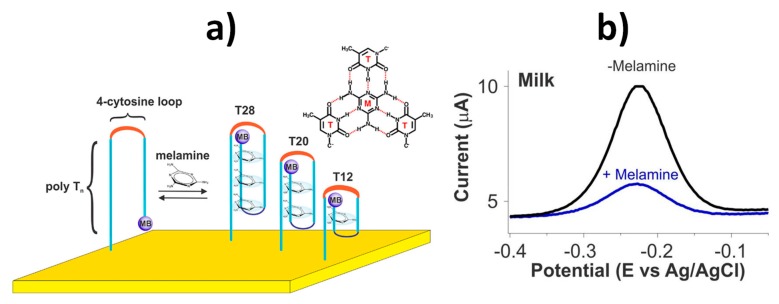
(**a**) Electrochemical switch-based sensor developed for melamine monitoring using a family of redox reporter (methylene blue, MB) modified, thiol-anchored DNA sequences comprised of two polythymine segments connected by a four-cytosine loop. (**b**) SWVs obtained in the absence and presence of 500 μM melamine. Reprinted from [35], with permission.

The method reported for the detection of melamine in milk used switching-based electrochemical biosensors involving self-assembling of a thiolated redox (methylene blue, MB)-tagged DNA probe onto gold electrodes (Figure 8a). In the presence of melanin, a DNA triplex is formed which places the MB further away from the electrode and, consequently, a lower square wave voltammetry (SWV) oxidation response was measured (Figure 8b). This kind of biosensors, providing simple and fast molecular monitoring in the clinical field, are scarcely explored so far in the food safety field despite they may allow continuous, reagentless and almost real-time electrochemical biosensing of relevant analytes in static or flowing samples. In addition, switch-based electrochemical sensors show promising features to overcome biofouling, stability, and calibration challenging issues faced by electrochemical biosensing in particularly complex samples such as clinical and food matrices [60,61]. 

It is also important to mention that most of the technologies described in these last two sections can be easily adapted to the detection of other types of molecules such as toxins or nucleic acids by changing affinity ligands (e.g., aptamers, oligonucleotides), creating detection panels for food safety and for food-source identification that may be used for a plethora of interesting applications, such as verification of food origin, absence of contaminants confirmation, or support of dietary restrictions for religious purposes. 

## 5. General Considerations, Challenges to Face and Future Prospects 

Food allergies affect the quality of life of a significant percentage of the population and place a considerable burden on the world economy. To protect sensitized consumers from exposure to allergens, the development of reliable, accurate, highly sensitive and selective analytical methods is mandatory. Classical immunoassays and DNA-based methods fulfill this objective. However, electrochemical affinity biosensors have emerged in recent years as innovative and promising tools for rapid, simple and in any setting detection due to the possibility of transferring the technology involved to disposable and portable devices, which is of high interest to consumers, manufacturers and government agencies implied in food safety control.

Although still far from advances in the clinical field, electrochemical affinity biosensors have demonstrated excellent capabilities for the determination of analytes characteristic of the presence of allergens or adulterations in both animal and plant foods. The smart use of nanomaterials as electrode modifiers, MBs as solid supports of the affinity bioassays, the exploration of new bioreceptors and amplification strategies involving the use of multienzymatic reagents are decisive in imparting electrochemical biosensing unique features for the determination of food allergens and adulterants. The methods reported in the last two years have shown the ability to determine protein allergens or adulterants at the level of few ng mL^−1^ or mg Kg^−1^ as well as DNA fragments from characteristic genes at pM level (with no need for amplification of the target DNA) in raw and processed food samples of different nature and variable complexity.

Particular noticeable achievements include:

(i) The first biosensing electrochemical platforms for the detection of egg traces in raw or processed foods by targeting ovomucoid protein [44] as well as of tomato seeds by determining a specific region of the *Sola l 7* encoding gene [57].
(ii) The continuous detection of targets near real-time and in flowing samples using reagentless switch-based electrochemical biosensors (e.g., melamine in milk) [35].
(iii) The reliable multidetermination of 5 relevant allergens (gliadin, Ara h1, Cor a 1, casein, ovalbumin) in only 6 min using an affordable POC system integrating a disposable allergen extraction device and an electronic keychain reader [21].
(iv) The detection of specific fruit allergens (the nonspecific lipid transfer protein Sola l 7 in tomato seeds) by targeting characteristic DNA sequences directly in 100 ng gDNA [57].
(v) The detection of meat adulterations directly in raw mitochondrial lysates obtained from a raw meat (detection of horse meat in beef meat) with no need for extracting the genetic material beforehand and in only 75 min [37].
(vi) The detection of milk adulterations with milk of other animal origin or colostrum by targeting IgGs specific to cow, sheep and goat in only 30 min [43].

However, despite these advances and opportunities, the electrochemical biosensors developed in this field are still in their infancy and must continue to face many challenges. The sources of food allergies and the rates of incidence keep on growing due to the growth of the world population, requiring additional sources of protein and leading to globalization and the extensive import of new food to which we are first exposed. The worldwide appearance of new food allergens and/or adulterants also implies the need for specific bioreceptors for their detection.

Sample preparation, especially in solid samples, continues to be a challenge for fast, simple and in situ analysis, as the food analysis demands. Furthermore, taking into account that the solubility and/or integrity of dietary proteins/DNA can be affected by pH, heat or other treatments, the extraction protocols are difficult to generalize and must be optimized particularly for different matrices and targets. The possibility of detecting several allergens in the same sample and/or the same allergen in several samples at the same time is a priority to reduce costs and analysis time. 

The use of nanomaterials as electrode modifiers, nanocatalysts, nanoreporters and nanocarriers, the widespread use of synthetic and/or recombinant bioreceptors (aptamers, peptides, switched-based probes, phages, nanobodies, etc.) and the exploration of electrode modifiers that impart surface antifouling properties [62] are expected to provide new capabilities for applications in the food analysis field. Other capabilities, currently pursued in clinics, that are highly interesting also in the food allergens and adulterants analysis field include the development of no-wash [31], reagentless, calibration-free [60,61] electrochemical biosensors able to provide near real-time and accurate detection with minimal sample pretreatments and minimal handling by the end users. 

Regardless of the specific application, the potential, versatility to easily adapt to other identifiable allergens/adulterants and the advantages they have, meeting the demands of POC detection without sacrificing sensitivity and accuracy, augur a promising future for electrochemical biosensors to address the challenges of food safety surveillance. It is foreseen that these technologies will incur significantly into our future lives allowing a higher understanding of the influence of the complexes and multiple factors (individual, environmental, geographic, etc.) involved in food allergies. These advances will also allow a more rigorous analysis of food products providing a significant contribution to improve consumer protection, reduce accidental or intentional exposure to allergens/adulterants and identify new targets and possible deficiencies in our food supply chain.

It is also unquestionable that, in this field, we must pursue coordinated work between all actors involved (producers, retailers, scientists, clinicians, etc.), to identify the real needs of the sector, agree protocols and analyze and establish threshold values and international standards for food allergens and adulterations. All this will contribute significantly to the incursion of electrochemical biosensing technology into the market in the foreseeable future and to fully exploit the unique possibilities it offers for the benefit of all society and in the management of food safety.

## Figures and Tables

**Table 1:** Electrochemical immunosensing methodologies reported since 2017 for the determination of food allergens and adulterants.

Electrode	Methodology	Target Allergen/Adulterant (Food)	Detection Technique	Linear Range	LOD	Sample	Steps */Assay Time	Ref.
Allergens								
AuNPs-SPCE	Sandwich immunosensor involving a bDAb further conjugated with Strep-AP	Ara h 6 (Peanut)	LSV (3-IP+AgNO_3_)	1–100 ng mL^−1^	0.27 ng mL^−1^	Complex chocolate-based product containing peanut	3/3 h starting from CAb- AuNPs-SPCE	[40,54]
SPCE	Sandwich immunoassay implemented onto HOOC-MBs involving the same Ab unmodified as capture and labeled with HRP as detector	Ovomucoid (Egg)	Amperometry (H_2_O_2_/HQ)	0.3–25 ng mL^−1^	0.1 ng mL^−1^	Eggs, wheat flour and baked bread	1/30 min starting from CAb-HOOC-MBs	[44]
GCE	Crosslinking of gliadin on the surface of a collagen-modified GCE, catalyzed by transglutaminase and recognition with a specific anti-gliadin antibody	Gliadin (Gluten)	EIS [Fe(CN)_6_]^3-/4-^	5–20 mg L^−1^	-	-	4/4 h	[55]
SWCNTs-Au-Cr deposited silicon wafer	Direct immunoassay onto and CAb covalently immobilized onto 1-PBSE-modified SWCNTs	Ara h 1 (Peanut)	LSV	1–1000 ng mL^−1^	1 ng mL^−1^	-	1/30 min starting from the SWCNT-based biosensor	[56]
Au-SPEs (individual and array of 8 × SPEs)	Sandwich immunoassays implemented on the surface of Epoxy-MBs	Gliadin, Ara h1, Cor a 1, Casein, Ovalbumin/Gluten, peanuts, hazelnuts, milk, and eggs.	Chronoamperometry (H_2_O_2_/TMB)	-	Gliadin: 0.075 mg Kg^−1^ Ara h1: 0.007 mg Kg^−1^ Cor a 1: 0.089 mg Kg^−1^ Casein: 0.170 mg Kg^−1^ Ovalbumin: 0.003 mg Kg^−1^	Bread, milk, cereal, cookie, ice cream, burger, salads with dressing, pizza, beer and gluten-free menu items from restaurants	2/6 min starting from CAb-Epoxy-MBs	[21]
SPCE	Sandwich immunoassay implemented onto HOOC-MBs involving specific CAb and DAb and enzymatic labeling with an HRP-secondary Ab	Shrimp TPM (Shrimp)	Amperometry (H_2_O_2_/HQ)	Up to 218.7 ng mL^−1^	47 pg mL^−1^	Raw and cooked shrimp and squid samples	3/3 h starting from CAb-HOOC-MBs	[41]
CNFs/SPCE coupled to a paper immunoaffinity platform	Sandwich immunoassay implemented onto paper microzone	Gliadin (Gluten)	Amperometry (H_2_O_2_/HQ)	Up to 80 μg kg^−1^	Gliadin: 0.005 mg Kg^−1^	Flour samples (manioc, rice, gluten free, common wheat)	2/15 min starting from CAb-paper	[42]
Adulterants								
SPCE and SP4CEs	Sandwich immunoassays implemented onto HOOC-MBs involving the same Ab unmodified as capture and labeled with HRP as detector	Bovine, Ovine and Caprine IgGs (Milk)	Amperomety (H_2_O_2_/HQ)	Bovine IgGs: 2.6−250 ng mL^−1^, Ovine IgGs: 2.7−250 ng mL^−1^, Caprine IgGs 2.2−250 ng mL^−1^	Bovine IgGs: 0.74 ng mL^−1^, Ovine IgGs: 0.82 ng mL^−1^, Caprine IgGs 0.66 ng mL^−1^	Colostrum, raw, pasteurized and UHT milk samples	1/30 min starting from CAb-HOOC-MBs	[43]

AP: alkaline phosphatase; AuNPs: gold nanoparticles; CAb: capture antibody; CNFs: carbon nanofibers; DAb: detector antibody; EIS: electrochemical impedance spectroscopy; GCE: glassy carbon electrode; HQ: hydroquinone; HRP: horseradish peroxidase; 3-IP: 3-indoxyl phosphate; LSV: linear square voltammetry; MBs: magnetic beads; 1-PBSE: 1-pyrenebutanoic acid succinimidyl ester; SPCE: screen-printed carbon electrodes; SP4CEs: four working screen-printed electrodes; SWCNTs: single-walled carbon nanotubes; TMB: 3,3′,5,5′-Tetramethylbenzidine; TPM: tropomyosin. * Number of steps involved in the determination other than detection and sample preparation.

**Table 2:** Electrochemical nucleic acid-based biosensing methods reported since 2017 for the determination of food allergens and adulterants.

Electrode	Methodology	Target Allergen/Adulterant (Food)	Detection Technique	Linear Range	LOD	Sample	Steps */Assay Time	Ref.
Allergens								
SPCE	Sandwich hybridization assay involving biotinylated DNA Cp and Dp implemented on the surface of Strep-MBs and coupled to Express-PCR (100 bp-amplicon)	Cor a 9 (Hazelnut)	Amperometry (H_2_O_2_/HQ)	0.0024–0.75 nM (synthetic target DNA)	0.72 pM (synthetic target DNA) 20 pg gDNA extracted from hazelnut	hazelnut varieties and other species of similar families (pistachio, cashew, walnut and tangerine)	1/15 min starting from bCp-Strep-MBs	[36]
SPCE	Indirect competitive approach between a biotinylated peptide (b-Pep) immobilized onto Strep-SPCE and gluten proteins for a defined concentration of biotinylated Gli4 aptamer further labeling with Strep-HRP	Gliadin (Gluten)	Chronoamperometry (H_2_O_2_/TMB)	1–100 μg L^−1^ (four-parameter logistic equation)	0.113 μg L^−1^ Gliadin (< >380 μg kg^−1^ gluten)	Food samples (Fixamyl, Rolled oats, Fit Snack)	2/60 min starting from b-Pep-Strep-SPCE	[16]
SPCE	Sandwich hybridization assay involving a b-RNACp and a RNADp implemented on the surface of Strep-MBs and recognition of the RNA/DNA heterohybrids with Ab_RNA/DNA_ further conjugated with an HRP-secondary antibody	Sola l 7 (Tomato)	Amperometry (H_2_O_2_/HQ)	0.8–50 pM (synthetic target)	0.2 pM (synthetic target)	Tomato and corn (100 ng extracted gDNA)	2/1.5 h starting from b-RNACp-Strep-MBs	[57]
AuNPs-modified Au-SPE	Direct aptasensing approach at AuNPs-Au-SPE modified with a SH-Aptamer	Lysozyme	CV ([Fe(CN)_6_]^3-^)	1–10 μg mL^−1^	0.32 μg mL^−1^	Wines	1/1 h starting from SH-Aptamer-AuNPs-Au-SPE	[33]
SPCE	Indirect competitive approach between a biotinylated peptide (b-Pep) immobilized onto Strep-MBs and gluten proteins for a defined concentration of biotinylated Gli1 aptamer further labeling with Strep-HRP	Gliadin (Gluten)	Chronoamperometry (H_2_O_2_/TMB)	-	-	-	2/60 min starting from b-Pep-Strep-MBs	[34]
Adulterants								
SPCE	Direct hybridization assay at b-RNACp-Strep-MBs and and recognition of the RNA/DNA heterohybrids with Ab_RNA/DNA_ further conjugated with ProtA-polyHRP40	Specific fragment of horse mitochondrial DNA D-loop region (Meat)	Amperomety (H_2_O_2_/HQ)	0.39–75 pM	0.12 pM (synthetic target DNA)	Beef meat spiked with horse meat	2/1 h 30 min starting from b-RNACp-Strep-MBs	[37]
Au disk(2-mm)	E-DNA based on a MB-modified thiolated DNA (Signal-off)	Melamine (Milk)	SWV (MB)	-	150 μM(~19 ppm) in buffered solutions,20 μM (~2.5 ppm) in whole milk	Milk	Continuous, real-time mesurements in flowing samples	[35]

AuNPs: gold nanoparticles; b: biotin; Cp: capture probe; Dp: detector probe; HQ: hydroquinone; HRP: horseradish peroxidase; LSV: linear square voltammetry; MB: methylene blue; MBs: magnetic beads; Pep: peptide; SPCE: screen-printed carbon electrodes; Strep: streptavidin; SWV: square wave voltammetry; TMB: 3,3′,5,5′-Tetramethylbenzidine. * Number of steps involved in the determination other than detection and sample preparation.
